# Acute kidney injury following household pyrethroid aerosol insecticide exposure: a case report and literature review

**DOI:** 10.3389/fmed.2026.1805866

**Published:** 2026-05-28

**Authors:** Yuxin Tang, Yuqing Wu, Xuhang Xue, Sihan Tao, Xing Tao, Li Li, Lianyi Geng, Kun Gao, Xufang Wang

**Affiliations:** 1 Department of Nephrology, Affiliated Hospital of Nanjing University of Chinese Medicine, Nanjing, China; 2 Jiangsu University Key Laboratory of Tonifying Kidney and Anti-senescence, Nanjing University of Chinese Medicine, Nanjing, China; 3 Department of Pathology, Affiliated Hospital of Nanjing University of Chinese Medicine, Nanjing, China; 4 Division of Emergency Medicine, Affiliated Hospital of Nanjing University of Chinese Medicine, Nanjing, China

**Keywords:** acute kidney injury, pyrethroids, acute interstitial nephritis, acute tubular necrosis, nephrotoxicity

## Abstract

**Background:**

Pyrethroids (PYRs) are increasingly detected in both environmental matrices and human populations. Emerging evidence underscores their potential for systemic toxicity. Biopsy-confirmed nephrotoxicity in humans remains sparsely documented, particularly in non-occupational exposure. We present a case of acute kidney injury (AKI) associated with household pyrethroid-containing aerosol insecticide exposure.

**Case presentation:**

A 55-year-old previously healthy male presented with nausea and abdominal pain and was found to have AKI on laboratory evaluation. He denied prior medication use or history of drug allergies. Detailed history revealed that the patient had stayed overnight in a small, poorly ventilated bedroom that had been heavily sprayed with a household pyrethroid-containing aerosol insecticide earlier that evening. Despite receiving empirical treatment, the patient’s renal function progressively deteriorated. Kidney biopsy revealed acute tubular necrosis (ATN) together with acute interstitial nephritis (AIN). After corticosteroid therapy, his renal function partially improved within 3 weeks. During follow-up, renal function stabilized at stage 3 chronic kidney disease (CKD).

**Conclusion:**

The case suggests that inappropriate exposure to PYRs may be associated with clinically significant nephrotoxicity, a complication rarely described in humans. Clinicians should consider this environmental toxicant in the differential diagnosis of otherwise unexplained AKI. In addition, the literature review provides mechanistic insights into PYR-associated nephrotoxicity, particularly oxidative stress, inflammation, mitochondrial dysfunction, and tubular injury.

## Introduction

Pyrethroids (PYRs) are synthetic insecticides widely used in agriculture, public health, and household pest control. PYRs are generally considered safe because of their relatively low mammalian toxicity. However, non-occupational exposure has become increasingly common due to their extensive domestic use and may pose serious health risks (1). PYRs can enter the human body through inhalation, dermal contact, or ingestion. Pyrethroid metabolites have been detected in the urine of the general population, even including pregnant women and children (2). Accumulating evidence suggests that substantial or inappropriate PYRs exposure have adverse effects on multiple organs, including the reproductive, immune, respiratory, and nervous systems (3).

Human evidence linking PYR exposure to renal injury remains limited. In the present report, we describe a case of acute kidney injury (AKI) probably associated with intense household exposure to a pyrethroid-containing aerosol insecticide. Kidney biopsy confirmed a diagnosis of acute tubular necrosis (ATN) coexisting with acute interstitial nephritis (AIN). This case adds histopathological evidence suggesting the potential nephrotoxicity of PYR-containing insecticides, particularly following substantial exposure in a poorly ventilated environment.

## Case presentation

A 55-year-old previously healthy man was referred to the Affiliated Hospital of Nanjing University of Chinese Medicine on November 29, 2024, with acute kidney injury (AKI). He had no history of renal insufficiency or chronic illness. Likewise, he reported no prior medication use or allergies. Three weeks before admission, he stayed at a relative’s home for 5 days. The bedroom in which he slept was approximately 10 m^2^. Because the bedroom windows had been left open earlier, mosquitoes were present in the room. The room was subsequently heavily sprayed with a household pyrethroid-containing aerosol insecticide in the late afternoon or early evening. According to the product label, the aerosol contained 0.16% active pyrethroid ingredients, including cypermethrin 0.10%, prallethrin 0.03%, and imiprothrin 0.03%. The remaining components were described as suitable solvents and propellants. The family estimated that the room was sprayed for approximately 1 to 2 min in the late afternoon or early evening. After spraying, the windows were closed, and the room was not ventilated. The patient entered the room after dinner and slept in this room for five consecutive nights. A strong insecticide odor was noticeable on the bedding during the night. Other family members did not sleep in this room and remained asymptomatic. He subsequently developed nausea and abdominal pain, prompting medical evaluation. Initial tests at a local hospital revealed an elevated high-sensitivity C-reactive protein of 34.92 mg/L and white blood cell count of 12.95 × 10^9^/L with predominant neutrophilia, whereas lymphocyte and eosinophil counts were within normal ranges. Liver function was unremarkable, with alanine aminotransferase 6 IU/L, aspartate aminotransferase 13 IU/L, total protein 87.7 g/L and albumin-to-globulin ratio 1.08. Renal function was impaired, with serum creatinine (Scr) of 290 μmol/L, blood urea nitrogen (BUN) of 10.58 mmol/L and an estimated glomerular filtration rate (eGFR) of 20.15 mL/min/1.73 m^2^. After AKI had been identified, the patient then received empirical treatment with a penicillin-class antibiotic, a proton pump inhibitor (PPI) and an antispasmodic. His abdominal symptoms improved, but renal function worsened over the following 3 days, with Scr and BUN elevated to 375 μmol/L and 12.46 mmol/L respectively, and eGFR dropping to 14.77 mL/min/1.73m^2^. Urinalysis showed proteinuria (+), glucosuria (+++), with 16 WBC/μL. Renal ultrasound revealed normal kidney morphology. The patient was referred to our hospital the following day.

Upon admission, his vital signs were within normal limits except for elevated blood pressure (158/95 mmHg). Laboratory tests showed persistent inflammation and worsening renal dysfunction, with Scr 438.9 μmol/L and BUN 11.2 mmol/L. The patient remained non-oliguric throughout hospitalization. His daily urine output ranged from 800 mL to 3,200 mL, despite the progressive elevation of Scr. Serum electrolytes were largely unremarkable, except for mild metabolic acidosis with bicarbonate of 18.6 mmol/L. Liver function tests remained within normal ranges. Evidence of tubular injury was indicated by markedly elevated urinary tubular markers, including retinol-binding protein 92.87 mg/L, α1-microglobulin 167.9 mg/L and a microalbumin-to-creatinine ratio (uACR) 195 mg/g. Urinalysis continued to show proteinuria (+) and glycosuria (++++). Additional laboratory investigations were negative, including antinuclear antibodies (ANA), C3 and C4 complement levels, serum immunoglobulin levels, serum and urine protein electrophoresis with immunofixation, anti–glomerular basement membrane antibodies, and antineutrophil cytoplasmic antibodies, arguing against immune-mediated glomerular diseases.

**Figure 1:**
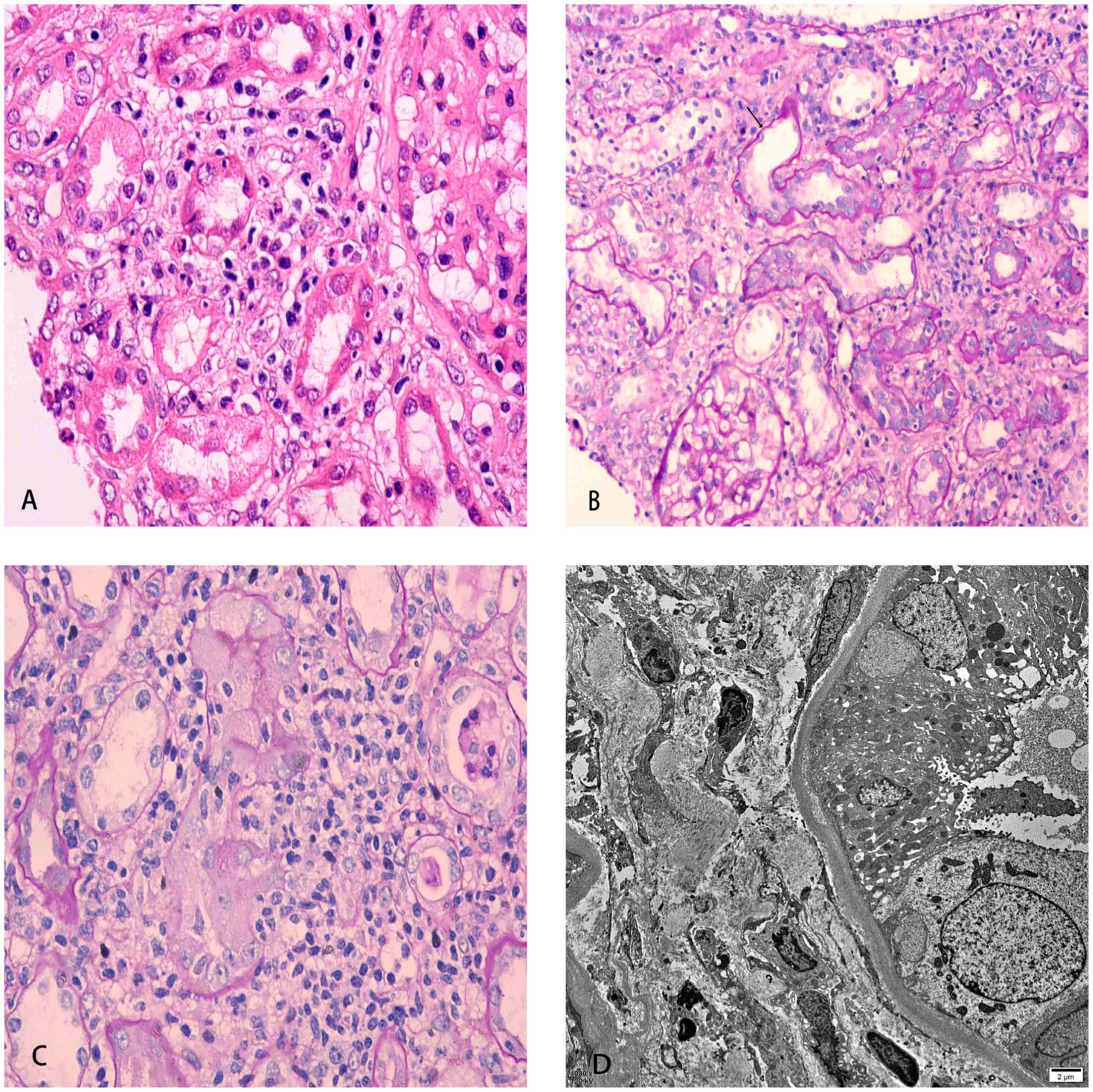
Kidney biopsy findings. **(A)** Extensive interstitial infiltration of inflammatory cells, predominantly lymphocytes, with scattered neutrophils and eosinophils (original magnification, ×400). **(B)** Light microscopy showing acute tubular injury with loss of brush border and areas of bare basement membrane (original magnification, ×200). **(C)** Swollen tubules with tubular basement membrane breaks, inflammatory cell infiltration, and histiocytic reaction (original magnification, ×400). **(D)** Electron microscopy showing intact glomeruli and minimal interstitial inflammatory infiltration (original magnification, ×4,000).

To clarify the cause of rapidly progressive renal impairment, a kidney biopsy was performed. Light microscopy revealed 14 glomeruli with preserved architecture and no abnormalities. The tubulointerstitial compartment showed dense infiltration with lymphocytes, neutrophils and occasional eosinophils. Approximately 60% of kidney tubules exhibited acute injury, characterized by epithelial cell flattening, loss of brush border and some bare membrane changes. Tubular swelling, focal disruption of the tubular basement membrane (TBM), inflammatory cell infiltration, histiocytic reaction, and tubulitis were also observed. Multifocal tubular atrophy and interstitial fibrosis (approximately 10%) were also observed (Figures 1A–C). Electron microscopy demonstrated inflammatory cells were seen in the renal interstitium (Figure 1D). No immune complex deposition was detected by either immunofluorescence or electron microscopy. Overall, the pathological findings were consistent with acute tubular necrosis (ATN), compatible with toxic tubular injury, together with acute interstitial nephritis (AIN).

**Figure 2:**
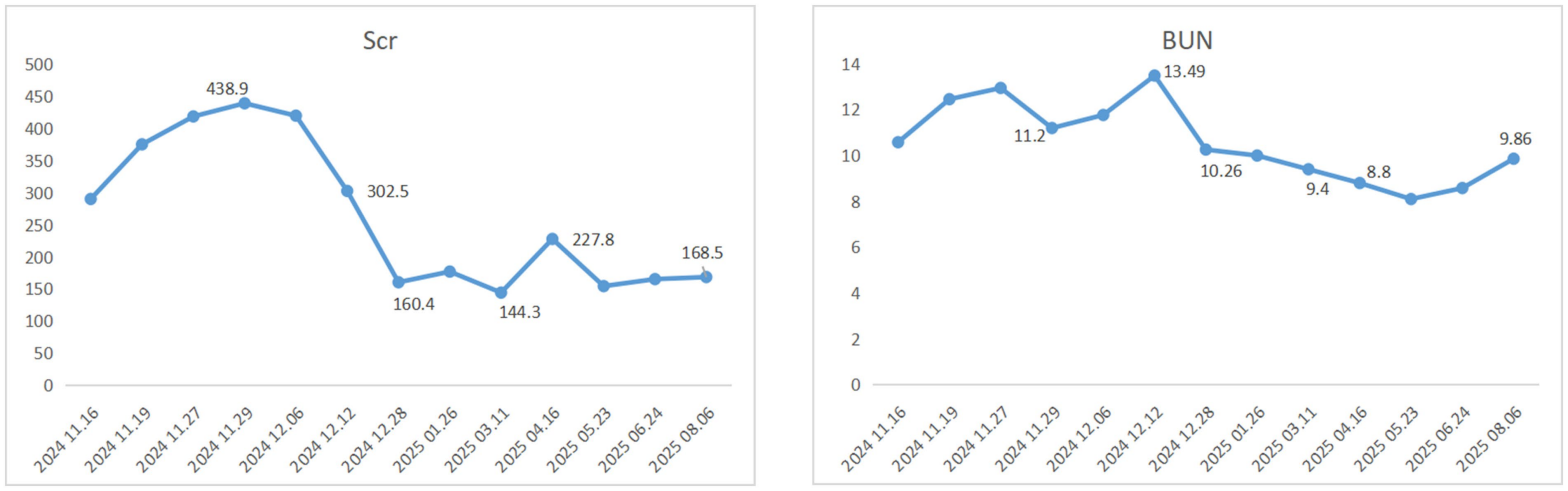
Trends in Scr (μmol/L) and BUN (mmol/L).

The patient was treated with intravenous methylprednisolone (40 mg/day) for six consecutive days, along with fluid resuscitation. This was followed by oral prednisolone (30 mg/day) for 2 weeks. Renal function progressively improved during hospitalization. By January 2025, the patient was maintained on prednisolone 25 mg/day. In March 2025, he self-discontinued corticosteroid therapy due to herpes zoster, resulting in a slight rebound increase in Scr. Prednisolone 10 mg/day was restarted on April 16 and tapered to 5 mg/day by August. At the 8-month follow-up, he exhibited persistent proteinuria (+) and stable Scr (144.3–227.8 μmol/L), consistent with stage 3 chronic kidney disease (CKD). Trends in Scr and BUN over time are summarized in Figure 2.

## Discussion

PYR-associated nephrotoxicity in humans may be underrecognized, particularly in non-occupational settings. Diagnosis is often challenging due to its nonspecific clinical manifestation. Exposure histories may be incomplete, and toxicological evidence is not routinely available. In this case, AKI had already been identified before the initiation of empirical pharmacotherapy. However, renal function continue to deteriorate after treatment with a penicillin-class antibiotic and a proton pump inhibitor. Kidney biopsy revealed a complex pattern of ATN coexisting with severe AIN. These findings suggest that both toxic tubular injury and immune-mediated tubulointerstitial inflammation should be considered in interpreting the renal pathology.

PYRs, derived from natural pyrethrins, are classified into type I and II based on chemical structure. The presence of an *α*-cyano group in Type II compounds contributes to their increased toxicity (4). PYRs have gradually replaced organophosphorus insecticides due to their better safety in humans (5). However, their lipophilic nature allows absorption via inhalation, ingestion or dermal contact (6). This may lead to systemic toxicity. Common symptoms of PYRs exposure include mild neurological and gastrointestinal symptoms such as nausea, vomiting, abdominal pain and dizziness (7). High-dose or intentional ingestion can result in severe complications. Increasing atypical presentations have been reported, including respiratory failure, complete heart block, AKI and seizures (8). Alveolar Hemorrhage (9), auditory hallucination (10) and myelopathy (11) have also been documented in exceedingly rare instances.

Reports of PYR-associated nephrotoxicity in humans remain rare but are gaining prominence. In a retrospective study involving 59 patients with PYRs poisoning, 39.3% presented with atypical features and AKI occurred in 10.7% of cases (8). Bashir, Babar et al. (12) reported a case of severe AKI in a 66-year-old woman following accidental inhalation, with biopsy-proven ATN. Given the prevalence of PYRs, renal injury may be underrecognized without histological confirmation. This case provides compelling pathological evidence for the complication.

As detailed in the Case presentation, the patient experienced repeated overnight exposure in a small, poorly ventilated bedroom after household aerosol insecticide spraying. The manufacturer’s standard specifies a spray rate of at least 2.0 g/s, with total active PYRs of 0.16%. Based on the family-estimated spraying duration of approximately 1–2 min, the theoretical release would be approximately 192–384 mg of total PYRs into the indoor environment. This estimate represents environmental release rather than the patient’s absorbed dose. The remaining majority of the formulation was described as suitable solvents, propellants, and other non-active formulation components, with liquefied petroleum gas specified as the propellant. Although the exact solvent composition was not fully disclosed, the active PYR ingredients remained the main toxicologically relevant insecticidal components.

Neither serum PYR compounds nor urinary metabolites, such as 3-phenoxybenzoic acid and DCVA, were measured, limiting direct toxicological confirmation (13). Reported toxic dose thresholds for PYRs are mainly derived from oral ingestion cases, with toxic oral doses of approximately 100–1,000 mg/kg body weight and potentially lethal oral doses estimated at 1–10 g (14). A recent review also suggests that acute oral toxicity may occur at approximately 100 mg/kg body weight or higher (15). However, these oral dose estimates cannot be directly extrapolated to repeated inhalational exposure in an enclosed indoor environment or to possible dermal exposure from contaminated bedding. Therefore, although the amount sprayed, room volume, airborne concentration, and absorbed dose could not be retrospectively quantified, the exposure history and semi-quantitative release estimate support exposure to a PYR-containing household aerosol insecticide. Further studies are needed to clarify dose–response relationships and biomarker thresholds for PYR-associated kidney injury.

Other potential causes of AKI and systemic diseases were carefully evaluated. Immune-mediated glomerular diseases and systemic vasculitis were unlikely because ANA, C3 and C4 complement levels, anti-GBM antibodies, ANCA, serum immunoglobulin levels, and serum and urine protein electrophoresis with immunofixation were unremarkable. Kidney biopsy showed preserved glomerular architecture without immune complex deposition, and renal ultrasonography did not support obstructive nephropathy.

The contribution of empirical medications should also be considered. Penicillin-class antibiotics and PPIs are recognized triggers of drug-induced AIN. However, AKI had already been identified before these medications were administered, with Scr reaching 290 μmol/L at the local hospital. Renal function continued to deteriorate after exposure to these drugs, and kidney biopsy showed interstitial inflammation with occasional eosinophils and tubulitis, findings compatible with a hypersensitivity-mediated component. Given the coexistence of extensive acute tubular injury and severe interstitial inflammation, we considered a more complex pathogenic process in which substantial PYR-containing aerosol exposure may have acted as the initial toxic insult, whereas subsequent empirical medications may have served as superimposed aggravating factors. We propose that the initial PYR-induced injury served as a priming event. This makes the renal microenvironment become hypersensitive (16, 17). In this context, the immunological threshold for common nephrosensitizing agents (18) (such as penicillin and PPIs) was lowered, transforming them into a synergistic second hit.

Among the active ingredients, cypermethrin (CYP) has been frequently detected as an environmental residue and has shown nephrotoxic potential in experimental studies (19). Animal models have demonstrated that CYP exposure can induce dose-dependent renal tubular injury, including tubular epithelial degeneration, brush border loss, and inflammatory changes (20). In the present case, the marked acute tubular injury involving approximately 60% of tubules, characterized by epithelial flattening and brush border loss, was compatible with a toxic tubular insult after exposure to a PYR-containing aerosol insecticide.

**Figure 3:**
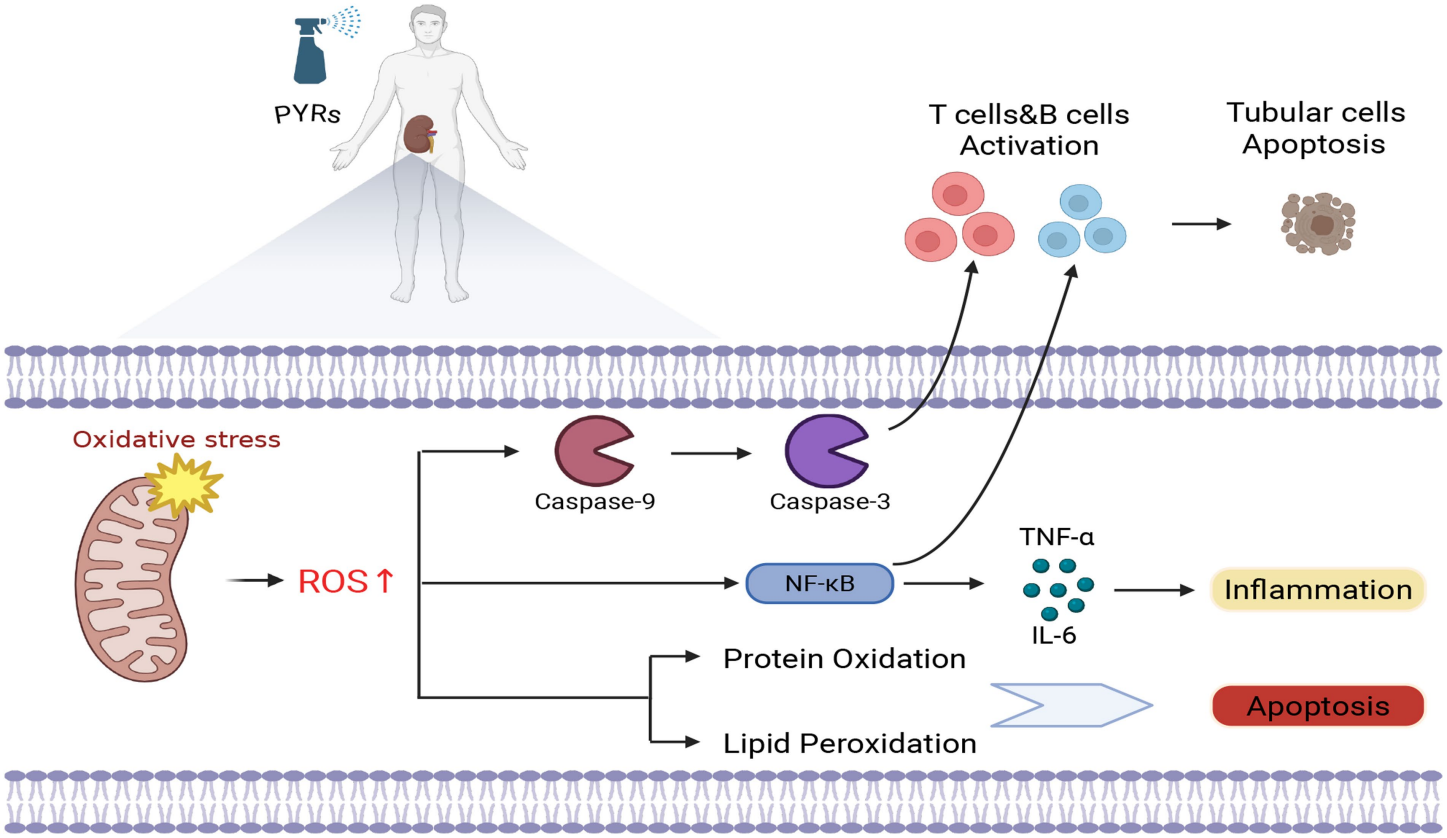
Proposed mechanisms of PYR-associated nephrotoxicity. PYRs may increase reactive oxygen species (ROS) production, leading to lipid, protein, and DNA damage. ROS can promote caspase-dependent tubular apoptosis and activate NF-κB signaling, thereby enhancing cytokine release and lymphocyte recruitment. These processes may collectively contribute to acute tubular injury and tubulointerstitial inflammation. Created in BioRender. Tang, Y. (2026) https://BioRender.com/ki8sje0. License: BioRender open-access publication license. Source: BioRender. The material was created/adapted using BioRender icons and schematic elements.

Oxidative stress is considered to be the central pathogenic mechanism of PYRs toxicity. PYRs exposure promotes the production of reactive oxygen species (ROS), which disrupt redox homeostasis and damage renal tubular epithelial cells (21). In CYP-treated rats, elevated level of malondialdehyde (MDA) and hydrogen peroxide (H₂O₂) were observed, along with reduced glutathione (GSH) level and catalase (CAT) activity in the renal tissue (22, 23). These reflect a disruption in redox defense system. Oxidative insults promote lipid peroxidation and DNA damage, ultimately leading to widespread ATN. This primary structural damage mediated by oxidative stress facilitated subsequent inflammation (24). ROS further activates intracellular signaling pathways, including NF-κB (25) and JNK/c-Jun (26), which enhance the release of pro-inflammatory cytokines such as TNF-*α* and IL-6. Oxidative stress and inflammatory synergistically trigger apoptosis via mitochondrial pathway (caspase-3/ caspase-9) (27) and death receptor pathway mediated by TNF-α (25, 28) (Figure 3). Autophagy and endoplasmic reticulum stress also contribute to PYR-induced cytotoxicity (29). Experimental evidence further reveals that direct PYR toxicity can cause severe tubular epithelial injury and disruption of the tubular basement membrane (TBM). This structural collapse reveals the massive release of Damage-Associated-Molecular Patterns (DAMPs) into the interstitium. These DAMPs and cytokines act as endogenous danger signals that trigger a necrotic inflammatory cascade, amplifying inflammatory responses (30). The combined effects of oxidative stress, inflammation and immune activation ultimately lead to tubular damage.

This case has several limitations. Serum PYR compounds and urinary metabolites were not measured, and the actual absorbed dose could not be reconstructed. The exact solvent composition was not fully disclosed, limiting assessment of the independent contribution of non-active formulation components. Nevertheless, the exposure history, clinical course, and histopathological findings collectively support a probable association between household PYR-containing aerosol exposure and kidney injury.

There is no specific antidote for PYR-associated AKI, and management primarily focuses on supportive care. Treatment should include prompt removal from exposure, adequate ventilation, fluid and electrolyte management, correction of acid–base disturbances, and close monitoring of renal function. Dialysis may be conducted if necessary. In patients with biopsy-proven AIN or strong suspicion of immune-mediated tubulointerstitial injury, corticosteroid therapy may be considered. Although bioactive compounds such as curcumin (23) and quercetin (29) have shown experimental protective effects by restoring antioxidant defenses and inhibiting inflammatory pathways, their clinical efficacy in PYR-associated AKI remains unproven.

Evidence regarding the long-term renal outcomes of PYRs exposure remains limited (31). In this case, the patient’s progression to stage 3 CKD 8 months post-AKI highlights the clinical challenge of such complex injuries. Although corticosteroid therapy attenuated the acute immunologically mediated inflammation (AIN), it was insufficient to reverse the established structural damage (ATN). The presence of renal tubular atrophy and interstitial fibrosis at the time of biopsy suggests maladaptive tubular repair—a pivotal mechanism in AKI-to-CKD transition (32). We consider the initial severity, coupled with the synergies of interstitial inflammation, acted as decisive risk factors. These ultimately led to permanent nephron loss and stabilized at stage 3 CKD.

## Conclusion

Environmental pollutants, including pesticides such as PYRs, are increasingly recognized as risk factors for kidney diseases. The nephrotoxicity of PYRs is well established. This case highlights the importance of considering environmental exposures in the differential diagnosis of acute kidney injury. Given the continued widespread exposure to PYRs, clinicians should be alert to the potential nephrotoxic effects. Public health education should emphasize safe household insecticide use, adequate ventilation, avoidance of excessive spraying, and delayed re-entry into treated rooms.

## Data Availability

The original contributions presented in the study are included in the article/supplementary material, further inquiries can be directed to the corresponding authors.
